# ERRγ Promotes Multiple Myeloma Survival by Coordinating NF-κB Signaling and Mitochondrial Apoptosis Regulation

**DOI:** 10.32604/or.2025.063700

**Published:** 2025-08-28

**Authors:** Xiaobing Zhou, Ying Li, Zizi Jing, Wei Yu, Jianbin Chen

**Affiliations:** 1 Department of Hematology, The First Affiliated Hospital of Chongqing Medical University, Chongqing, 400016, China; 2 Department of Hematology, University-Town Hospital of Chongqing Medical University, Chongqing, 400016, China

**Keywords:** Multiple myeloma (MM), estrogen-related receptor gamma (ERRγ), bone destruction, apoptosis, mitochondrial dysfunction, nuclear factor-kappa B (NF-κB)

## Abstract

**Background:**

Multiple myeloma (MM) remains a formidable clinical challenge due to its high relapse rate and resistance to existing therapies. Estrogen-related receptor gamma (ERRγ), a nuclear receptor critical for cellular energy metabolism, has been implicated in various cancers. but its role in MM remains unclear.

**Methods:**

ERRγ expression was assessed using bioinformatics and RT-qPCR. Functional studies were conducted through siRNA-mediated ERRγ knockdown and treatment with the inverse agonist GSK5182 to examine their effects on MM cell proliferation and apoptosis.

**Results:**

ERRγ was significantly upregulated in the bone marrow of MM patients, correlating with advanced clinical stages and pathological fractures. Inhibition of ERRγ reduced MM cell expansion both *in vitro* and *in vivo*, while promoting mitochondrial-dependent apoptosis. Co-immunoprecipitation assays demonstrated a physical association between ERRγ and P65. Inhibition of ERRγ attenuated canonical nuclear factor-kappa B (NF-κB) signaling by blocking the nuclear translocation of its key effector p65. Additionally, modulation of ERRγ altered receptor activator of nuclear factor-κB ligand (RANKL) levels, implying a potential role in bone degradation observed in MM cases.

**Conclusion:**

Collectively, the data broaden understanding of ERRγ’s contribution to MM development and propose it as a viable target for therapeutic intervention.

## 1 Introduction

Multiple myeloma (MM) is the second most common hematologic malignancy [1], marked by unchecked expansion of monoclonal plasma cells within the bone marrow, which results in bone degradation, impaired kidney function, anemia, and elevated calcium levels [2]. The introduction of therapies such as proteasome inhibitors, immunoregulators, monoclonal antibodies aimed at surface antigens, and autologous stem cell transplantation (ASCT) has greatly advanced outcomes for MM patients. Nonetheless, most individuals eventually experience disease relapse due to resistance to treatment, emphasizing an urgent demand for new biologically driven therapeutic strategies.

ESRRG (Estrogen-related receptor gamma, ERRγ) belongs to the orphan nuclear receptor family and lacks a confirmed natural ligand. It is abundantly present in tissues like the placenta, brain, skeletal muscles, heart, and liver [3]. Growing research points to its significant function in mitochondrial creation and the maintenance of cellular energy balance, where it adjusts metabolic responses during normal physiological states [4,5]. Apart from its metabolic duties, ERRγ also plays a cancer-promoting role in malignancies like small-cell lung cancer (SCLC), hepatocellular carcinoma (HCC), breast cancer, and retinoblastoma, aiding in resistance to therapy and survival under low-oxygen conditions [6–8]. In the sphere of bone biology, ERRγ has been shown to influence osteoblast formation by suppressing the activity of Runx2 through transcriptional mechanisms [9]. Targeting ERRγ pharmacologically has demonstrated promise by reducing osteoclast function and triggering programmed cell death [10]. However, its role in MM, a cancer typified by bone erosion and the clonal growth of plasma cells, has not been previously investigated.

The malignant persistence of MM cells is tightly linked to constitutive activation of nuclear factor-kappa B (NF-κB) signaling. Genomic profiling reveals that over 45% of newly diagnosed patients harbor mutations directly activating NF-κB pathways, which drive tumor survival, drug resistance, and clonal evolution [11–13]. Beyond cell-autonomous effects, NF-κB hyperactivation remodels the bone marrow niche by upregulating IL-6 and MCL1 to sustain MM proliferation while stimulating receptor activator of nuclear factor-κB Ligand (RANKL) secretion to promote osteoclast-mediated bone destruction [14]. Notably, ERRγ has been implicated in both direct and indirect crosstalk with NF-κB signaling across diverse pathological contexts, though its regulatory role appears highly cell type-dependent [8,15]. However, whether such interplay governs MM progression remains unexplored.

Therefore, this study aimed to delineate the expression profile and pathogenic role of ERRγ in MM, with a focus on its dual regulation of mitochondrial apoptosis and canonical NF-κB signaling through direct interaction with p65. We further identify clinical correlations between ERRγ overexpression and osteolytic bone lesions. These findings shed new light on the molecular mechanisms driving MM progression and suggest that targeting ERRγ may offer a novel therapeutic approach.

## 2 Materials and Methods

### 2.1 Microarray Data

RNA-seq data from 844 MM plasma cell samples, isolated with anti-CD138 magnetic beads, were obtained from the Multiple Myeloma Research Foundation (MMRF) CoMMpass cohort via the MMRF database (https://research.themmrf.org). Additional gene expression profiles (GSE118985, GSE6477, GSE16558, GSE755, GSE2658, and GSE13591) were sourced from the NCBI-GEO repository (http://www.ncbi.nlm.nih.gov/geo (accessed on 19 January 2025)). Datasets GSE6477, GSE16558, and GSE13591 include CD138-purified bone marrow plasma cells from newly diagnosed MM patients alongside healthy donor samples. GSE118985 comprises bone marrow biopsy specimens from 460 newly diagnosed MM patients and 68 healthy controls. GSE755 provides samples from 36 individuals without MRI-detected lytic lesions and 137 individuals presenting MRI-confirmed lytic lesions. GSE2658 features 559 CD138-selected MM plasma cell samples.

### 2.2 Patient Samples

To assess variations in ERRγ expression between MM cases and non-tumorous controls, bone marrow samples were collected from 55 newly diagnosed MM patients and 21 individuals with iron-deficiency anemia (IDA). Bone marrow mononuclear cells (BMMCs) were isolated using a Ficoll density-gradient centrifugation protocol with a human bone marrow lymphocyte isolation kit (Solarbio, P7250, Beijing, China). To better correlate ERRγ expression with clinical and pathological features, an additional cohort of 45 newly diagnosed MM patients was recruited, from whom plasma cells purified by anti-CD138 magnetic beads (>95% purity, Miltenyi Biotec, 130-097-614, Bergisch Gladbach, Germany) were isolated. All collected BMMCs and purified plasma cells were suspended in 1 mL AG RNAex Pro Reagent (Accurate Biology, AG21102, Changsha, China) and preserved at –80°C for subsequent analyses. Sample collection occurred between April 2023 and October 2024. All participants provided written informed consent, and the study received approval from the Ethics Committee of The First Affiliated Hospital of Chongqing Medical University (K2023-463). The research involving human specimens complied with the principles outlined in the Declaration of Helsinki. Individuals who had previously undergone radiotherapy, chemotherapy, treatment with immunoregulators or proteasome inhibitors, or autologous stem cell transplantation were excluded from the study.

### 2.3 Cell Culture

Human MM cell lines U266 and KMS11 were purchased from Procell Life Science and Technology Co., Ltd. (Wuhan, China). RPMI8226 was purchased from Shanghai Cell Bank of the Chinese Academy of Sciences (Shanghai, China). The MM1.S line was obtained from the Beijing Cell Bank of the Chinese Academy of Sciences (Beijing, China). OPM2 cells were a gift from Professor Jun Rao (Second Affiliated Hospital of Army Military Medical University, Chongqing, China). All cell lines were maintained in RPMI-1640 medium (Procell, PM150110, Wuhan, China) supplemented with 10% fetal bovine serum (PAN, ST30-3302, Aidenbach, Germany), 100 μg/mL streptomycin, and 100 U/mL penicillin (Beyotime, C0222, Shanghai, China) at 37°C under a 5% CO_2_ atmosphere. Media were refreshed every 2–3 days depending on cellular growth. All cell lines were tested for mycoplasma contamination and confirmed to be negative.

### 2.4 Quantitative Real-Time PCR

Total RNA was isolated using AG RNAex Pro Reagent (Accurate Biology, AG21102) according to the manufacturer’s instructions. RNA quantity and purity were determined with a NanoDrop 2000 spectrophotometer (Thermo Fisher Scientific, Waltham, MA, USA). For cDNA synthesis, 1 μg of RNA was reverse-transcribed utilizing the Evo M-MLV RT Mix Kit with gDNA Clean for qPCR Ver.2 (Accurate Biology, AG11728, Changsha, China). Quantitative real-time PCR was conducted using the SYBR Green Premix Pro Taq HS qPCR Kit (Accurate Biology, AG11701) on a CFX96 Real-Time PCR system (Bio-Rad Laboratories, Inc., Hercules, CA, USA). GAPDH served as the internal control, and relative gene expression was determined by the 2^–ΔΔCq^ method. Primer sequences used were:

Human GAPDH: forward 5^′^-GGAGTCCACTGGCGTCTTCA-3^′^, reverse 5^′^-GTCATGAGTCCTTCCACGATACC-3^′^.

Human ERRγ: forward 5^′^-CTACCCTTCTGCTCCTATCCTG-3^′^, reverse 5^′^-AGCGATGTCACCACACACTAAA-3^′^.

### 2.5 Small Interfering RNA (siRNA) Transfection

MM cells were transfected with siRNA targeting ERRγ or a negative control siRNA (si-NC) using the CALNP^TM^ RNAi *in vitro* Kit (D-Nano Therapeutics, Beijing, China) as per the manufacturer’s protocol, at a final concentration of 50 nM. The si-ERRγ sequence was designed by converting the validated shRNA sequence (5^′^-CCTGTCAGGAAACTGTATGAT-3^′^) from prior studies [6,16] into siRNA format (Sense: 5^′^-CCUGUCAGGAAACUGUAUGAU-3^′^; Antisense: 5^′^-AUCAUACAGUUUCCUGACAGG-3^′^). The negative control siRNA (si-NC) sequences were as follows: Sense: 5^′^-UUCUCCGAACGUGUCACGUTT-3^′^; Antisense: 5^′^-ACGUGACACGUUCGGAGAATT-3^′^.

### 2.6 Cell Viability Assay (CCK-8)

MM1.S, OPM2, and RPMI8226 cells were seeded at a density of 1 × 10^4^ cells per well in 96-well plates. Each well was supplemented with 90 μL of culture medium and 10 μL of CCK-8 reagent (Sparkjade, CT0001-B, Jinan, China). Following a 2-h incubation at 37°C, absorbance was recorded at 450 nm using a microplate reader (Thermo Fisher Scientific, MA, USA).

### 2.7 Nuclear and Mitochondrial Protein Isolation

Cytoplasmic, mitochondrial, and nuclear proteins were extracted sequentially using specific commercial kits. For mitochondrial and cytoplasmic separation, approximately 2 × 10^7^ cells were processed with the Cell Mitochondria Isolation Kit (Beyotime, C3601). Cells were first washed in ice-cold PBS, then suspended in 1 mL of isolation buffer and homogenized using a glass Dounce homogenizer. The homogenates were spun at 600× *g* for 10 min to remove nuclei and intact cells. The resulting supernatant was centrifuged again at 11,000× *g* for 10 min at 4°C, allowing collection of the cytoplasmic fraction (supernatant) and mitochondrial fraction (pellet). Mitochondrial proteins were dissolved in a lysis solution containing 1 mM Phenylmethylsulfonyl Fluoride (PMSF) (Beyotime, ST506).

To isolate nuclear proteins, 1 × 10^7^ RPMI8226 and OPM2 cells were processed with the Nuclear and Cytoplasmic Protein Extraction Kit (Beyotime, P0027). Cells were washed twice with PBS, then suspended in 200 μL Cytoplasmic Extraction Reagent A containing PMSF and vortexed strongly for 5 s, followed by a 15-min incubation on ice. After adding 10 μL of Cytoplasmic Extraction Reagent B, samples were vortexed (5 s), placed on ice for 1 min, vortexed again (5 s), and centrifuged at 12,000× *g* for 5 min at 4°C. The supernatant containing cytoplasmic proteins was collected, while nuclear pellets were lysed using radioimmunoprecipitation assay (RIPA) buffer (Beyotime, P0013D) supplemented with PMSF.

Protein concentrations from mitochondrial, cytoplasmic, and nuclear fractions were measured using the bicinchoninic acid (BCA) protein assay (Beyotime, P0010).

### 2.8 Western Blot Analysis

Proteins were extracted from MM cells using RIPA buffer (Beyotime, P0013D) and quantified using the BCA Protein Assay Kit. Equal amounts of protein (20–30 μg) were separated via SDS-PAGE and transferred to polyvinylidene difluoride (PVDF) membranes (Merck Millipore, IPVH00010, Burlington, MA, USA). The membranes were blocked with 5% nonfat milk for 2 h and incubated overnight at 4°C with primary antibodies, including: Histone H3 (ABclonal, A21107, 1:1000, Wuhan, China), ERRγ (Huabio, ER61910, 1:1000, Hangzhou, China), Cleaved caspase-3 (Huabio, ET1608-64, 1:1000), BCL2 (Huabio, ET1702-53, 1:1000), RANKL (Huabio, HA500369, 1:1000), β-tubulin (Huabio, ET1602-4, 1:5000), NF-κB p105/p50 (Huabio, ET1603-18, 1:5000), VDAC1 (Proteintech, 66345-1-Ig, 1:5000, Wuhan, China), BAX (Proteintech, 50599-2-Ig, 1:8000), β-actin (Proteintech, 66009-1-lg, 1:5000), Phospho-IκB alpha (ser36) (Selleck, F2237, 1:1000, Houston, TX, USA), IκB alpha (Wanlei Biotechnology, WL01936, 1:500, Hangzhou, China), γH2AX (Huabio, ET1602-2, 1:1000), Cytochrome c (Huabio, ET1610-60, 1:2000), NF-κB p65 (Abmart, T55034, 1:5000, Shanghai, China), Phospho-NF-κB p65 (Abmart, TP56372, 1:1000), Phospho-NF-κB p105/p50 (ZenBio, 310171, 1:1000, Chengdu, China). After washing, membranes were incubated with HRP-conjugated secondary antibodies (1:5000, Proteintech) for 1 h at room temperature. Signals were detected using an enhanced chemiluminescence (ECL) kit (Beyotime, P0018S) and visualized with the ChemiDoc^TM^ imaging system (Bio-Rad Laboratories, Hercules, CA, USA). Band intensities were quantified using ImageJ v1.54d software (National Institutes of Health, USA).

### 2.9 Co-Immunoprecipitation (Co-IP)

To investigate protein-protein interactions between ESRRG and NF-κB p65, Co-IP assays were performed. Briefly, 1 × 10^7^ RPMI8226 and OPM2 cells were lysed in Western and IP lysis buffer (Beyotime, P0013J) supplemented with 1 mM PMSF (Beyotime, ST506) for 30 min on ice. After centrifugation at 12,000× *g* for 15 min at 4°C, supernatants were divided into three groups: Input, IgG control, and IP. The IgG control group received 2 μL Rabbit IgG (Huabio, PSH04-02), while the IP group was incubated with 2 μL anti-p65 antibody (Huabio, ET1603-12, 1:1000) for 6 h at 4°C. Subsequently, 40 μL Protein A/G Magnetic Beads (Selleck, B23202, Houston, TX, USA) were added to each group, followed by overnight rotation at 4°C. Beads were washed three times with 0.05% PBST (Servicebio, G2157-1L, Wuhan, China) and resuspended in 60 μL 1× SDS-PAGE loading buffer (Beyotime, P0015). Samples were boiled at 95°C for 5 min and subjected to Western blot analysis. For Western blot detection, anti-rabbit IgG (H + L) secondary antibody (Huabio, M1208-2, 1:5000) was used to avoid interference from heavy chains.

### 2.10 Animal Studies

All experimental procedures followed the AVMA Guidelines for the Euthanasia of Animals (2020) and received approval from the Institutional Animal Care and Use Committee of Chongqing Medical University (Approval# IACUC-CQMU-2025-02071). Female Non-Obese Diabetic-Severe Combined Immunodeficiency (NOD-SCID) mice aged 4–5 weeks were obtained from Chengdu GemPharmatech Co., Ltd. (Chengdu, China) and maintained under Specific Pathogen-Free (SPF) conditions at Chongqing Medical University. Mice were randomly assigned into two groups (n = 4 per group) and subcutaneously inoculated with 5 × 10^6^ MM1.S cells into the right dorsal flank. Once tumors grew to approximately 100 mm^3^, the treatment group was given GSK5182 (40 mg/kg/day, intraperitoneally) for 14 days, while the control group received the same volume of vehicle solution. Tumor volumes (calculated as length × width^2^/2) and body weights were monitored every three days. On day 15, mice were euthanized by CO_2_ inhalation (flow rate: 20% of the chamber volume per minute), followed by cervical dislocation for death confirmation. Tumors were then excised, weighed, and processed for further studies.

### 2.11 Flow Cytometry for Apoptosis

Apoptotic rates were evaluated using the Annexin V-APC/PI Apoptosis Kit (MULTI SCIENCES, AP107, Hangzhou, China) according to the supplier’s protocol. Cells treated with siRNA or exposed to GSK5182 (MedChemExpress, HY-111226, Monmouth Junction, NJ, USA) for 48 h were harvested, rinsed with PBS, and stained with 5 μL Annexin V-APC and 10 μL propidium iodide (PI). After a 5-min incubation at room temperature, samples were assessed with a BD FACSCalibur flow cytometer (Becton, Dickinson and Company, Franklin Lakes, NJ, USA), and data were processed using FlowJo 8.6.3 software (Tree Star, Inc.).

### 2.12 Measurement of Mitochondrial Membrane Potential (ΔΨm)

Mitochondrial membrane potential was determined with the Mitochondrial Membrane Potential Assay Kit using JC-1 dye (MeiLunBio, MA0338_A, Dalian, China) according to the manufacturer’s protocol. Cells were incubated with JC-1 working solution for 20 min at 37°C, then washed twice with PBS before being analyzed with a Navios flow cytometer (Beckman Coulter Life Sciences, Brea, CA, USA). Data analysis was performed using FlowJo 8.6.3 software (Tree Star, Inc.).

### 2.13 Reactive Oxygen Species (ROS) Assay

Intracellular ROS production was assessed using the Reactive Oxygen Species Assay Kit (Solarbio, CA1410, Beijing, China) following the manufacturer’s guidelines. Cells were incubated with 2^′^,7^′^-Dichlorodihydrofluorescein diacetate (DCFH-DA), diluted 1:20,000 in culture medium, for 30 min at 37°C. Following three PBS washes, ROS fluorescence was measured using a Navios flow cytometer, and the resulting data were analyzed with FlowJo 8.6.3 software (Tree Star, Inc.).

### 2.14 Statistical Analysis

All statistical evaluations were carried out using GraphPad Prism v8.0 (GraphPad Software, San Diego, CA, USA). Data are expressed as the mean ± standard deviation (SD) from at least three separate experiments. Categorical variables were assessed using either Fisher’s exact test or the Chi-square test, while continuous variables were compared with Student’s *t*-test. Spearman’s correlation analysis was used to investigate the relationship between ERRγ and NF-κB pathway gene expression (NFKB1, NFKB2, RelA, and RelB). A *p* value less than 0.05 was regarded as statistically significant.

## 3 Results

### 3.1 ERRγ Is Upregulated in MM Patients and Correlates with Bone Destruction

**Figure 1:**
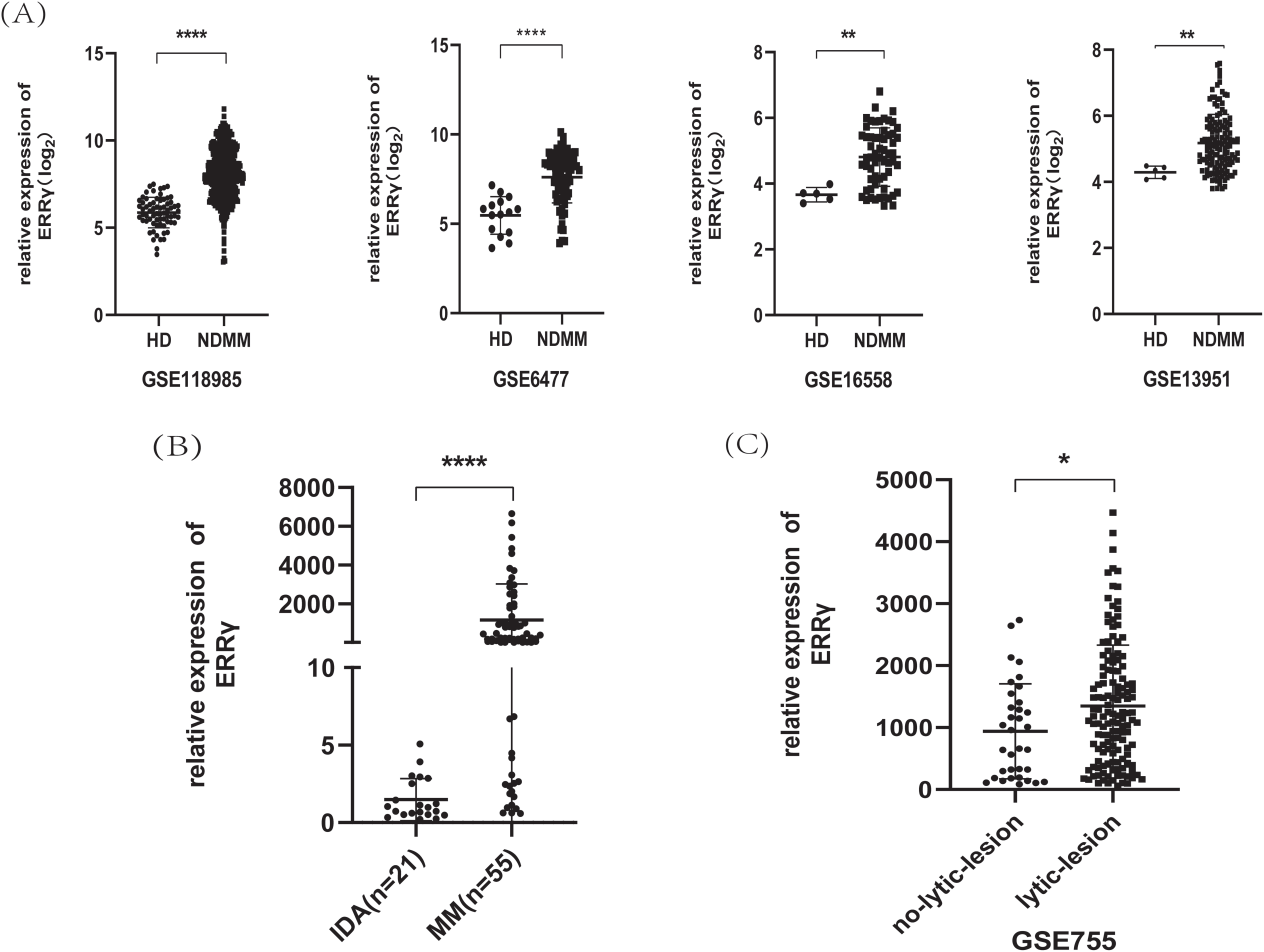
ERRγ expression is elevated in MM and linked to bone damage. **(A)** Analysis of GEO datasets showing increased ERRγ levels in MM patients compared to healthy donors. **(B)** RT-PCR confirmation of ERRγ expression in bone marrow samples from MM and IDA patients. **(C)** Higher ERRγ expression observed in MM patients with MRI-confirmed lytic bone lesions (GSE755). *p < 0.05, **p < 0.01, ****p < 0.0001

Analysis of GEO datasets (GSE6477, GSE13591, GSE16558, and GSE118985) revealed significantly higher ERRγ expression in newly diagnosed MM patients compared to healthy donors (Fig. 1A). Due to the difficulty in obtaining normal plasma cells, bone marrow mononuclear cells were collected from 21 IDA patients and 55 newly diagnosed MM patients. RT-PCR confirmed that ERRγ expression was significantly higher in MM patients (Fig. 1B).

To better understand the clinical implications of ERRγ, CD138-purified plasma cells were collected from 45 newly diagnosed MM patients, and ERRγ levels were correlated with clinical parameters. No significant association was observed between ERRγ expression and sex, age, creatinine, albumin, β2-microglobulin, or serum calcium; however, a strong link was found with Durie-Salmon staging (*p* = 0.0491). Strikingly, 82% of patients with elevated ERRγ expression presented with pathological fractures, compared to 30.4% of those with lower levels (*p* = 0.0005) (Table 1).

**Table 1:** Correlation analysis between ERRγ expression and clinical characteristics in 45 MM plasma cell samples

Characteristic	All cases	ERRγ expression		*p*-value
		Low (n = 23)	High (n = 22)	
Sex				0.9031
Male	27	14	13	
Female	18	9	9	
Age				0.0574
<60 (years)	14	4	10	
≥60 (years)	31	19	12	
Hemoglobin (g/L)				0.4651
<100	27	15	12	
≥100	18	8	10	
M protein				0.2478
IgG	20	8	12	
IgA	11	8	3	
IgM	1	1	0	
Light chain	13	6	7	
β2-MG (mg/L)				0.42
<5.5	28	13	15	
≥5.5	17	10	7	
Alb (g/L)				0.6085
<35	16	9	7	
≥35	29	14	15	
Cr (μmol/L)				0.7485
<177	37	19	18	
≥177	8	4	4	
Pathological fracture				0.0005***
Yes	25	7	18	
No	20	16	4	
Ca^2+^ (mmol/L)				0.7932
<2.65	34	17	17	
≥2.65	11	6	5	
DS staging system				0.0491*
I + II	5	5	0	
III	40	18	22	

Note: MM: multiple myeloma, M protein: monoclonal Protein, β2-MG: β2-microglobulin, Alb: albumin, Cr: creatinine, DS Staging System: Durie-Salmon Staging System, *p < 0.05, ***p < 0.001.

Additionally, analysis of the GSE755 dataset, which contains bone marrow plasma cells categorized by MRI-detected lytic lesions, revealed that after excluding the top 5% of outliers, ERRγ remained significantly higher in patients with bone lesions than in those without (Fig. 1C). Collectively, these findings suggest that ERRγ overexpression in MM is strongly associated with bone destruction.

### 3.2 Inhibition of ERRγ Suppressed MM Cells Proliferation and Induced Apoptosis

**Figure 2:**
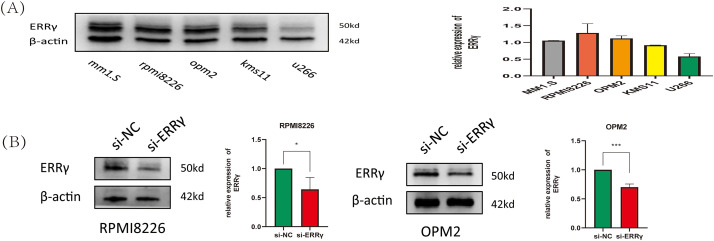

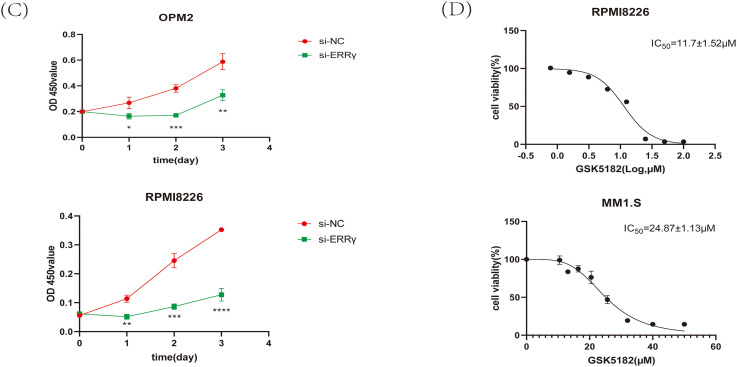
Suppression of ERRγ reduces MM cell proliferation. **(A)** Western blot showing ERRγ protein levels across five MM cell lines (MM1.S, RPMI8226, OPM2, KMS11, and U266). **(B)** Validation of reduced ERRγ protein levels in RPMI8226 and OPM2 cells 48 h after siRNA transfection. **(C)** CCK-8 assay demonstrating decreased cell viability in RPMI8226 and OPM2 following ERRγ knockdown. **(D)** Viability assay of MM1.S and RPMI8226 cells treated with different doses of GSK5182 for 48 h, with IC50 values calculated using GraphPad Prism 8. *p < 0.05, **p < 0.01, ***p < 0.001, ****p < 0.0001

The expression levels of ERRγ were assessed in five MM cell lines (MM1.S, RPMI8226, OPM2, KMS11, and U266) through Western blotting. Among these, MM1.S, RPMI8226, and OPM2 cells displayed high ERRγ expression and were chosen for further functional analyses (Fig. 2A). Validated small interfering RNA (siRNA) sequences targeting human ERRγ (5^′^-CCTGTCAGGAAACTGTATGAT-3^′^ [6,16]) were used to knock down ERRγ in MM cells. Western blot analysis confirmed a significant reduction in ERRγ protein levels in MM cells following siRNA-mediated knockdown (Fig. 2B).

To evaluate the impact of ERRγ inhibition on MM cell proliferation, cell viability was assessed using the CCK-8 assay. ERRγ knockdown led to a significant reduction in the proliferation of RPMI8226 and OPM2 cells (Fig. 2C). Similarly, GSK5182, an inverse agonist of ERRγ [17], reduced the viability of MM cells in a dose-dependent manner. The IC50 values for GSK5182 were determined to be 11.7 ± 1.52 μM for RPMI8226 cells and 24.87 ± 1.13 μM for MM1. S cells after 48 h of treatment (Fig. 2D). Based on these IC50 values, subsequent experiments were performed with 15 μM GSK5182 for RPMI8226 cells and 25 μM GSK5182 for MM1.S cells.

**Figure 3:**
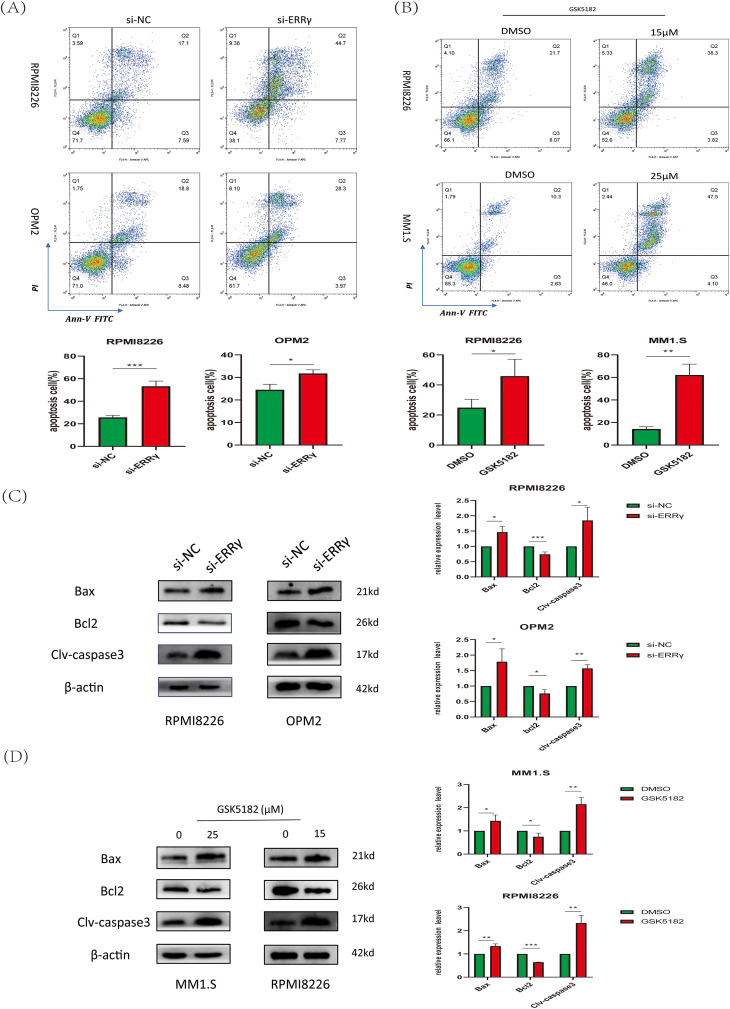
ERRγ inhibition induces apoptosis in MM cells. **(A)** Flow cytometric analysis showing increased apoptotic cells in RPMI8226 and OPM2 after ERRγ knockdown for 48 h. **(B)** GSK5182 treatment for 48 h significantly increased apoptosis in MM cells. **(C,D)** Western blot results showing elevated cleaved caspase-3 and BAX and reduced BCL2 levels in MM cells after 48 h of ERRγ inhibition. *p < 0.05, **p < 0.01, ***p < 0.001

Apoptosis was examined using Annexin V-FITC/PI dual staining followed by flow cytometry. A notable increase in apoptotic cells was observed in RPMI8226 and OPM2 cells after ERRγ knockdown compared to the scrambled control group (Fig. 3A). Similarly, pharmacological inhibition with GSK5182 induced apoptosis in MM1.S and RPMI8226 cells (Fig. 3B). Mechanistic studies showed that ERRγ inhibition elevated the levels of pro-apoptotic proteins (cleaved caspase-3 and BAX) and reduced the expression of the anti-apoptotic protein BCL2 across all tested MM cell lines (Fig. 3C,D). These findings suggest that both genetic and pharmacological targeting of ERRγ effectively triggers apoptosis in MM cells.

### 3.3 ERRγ Inhibition Suppresses MM Tumor Growth In Vivo

**Figure 4:**
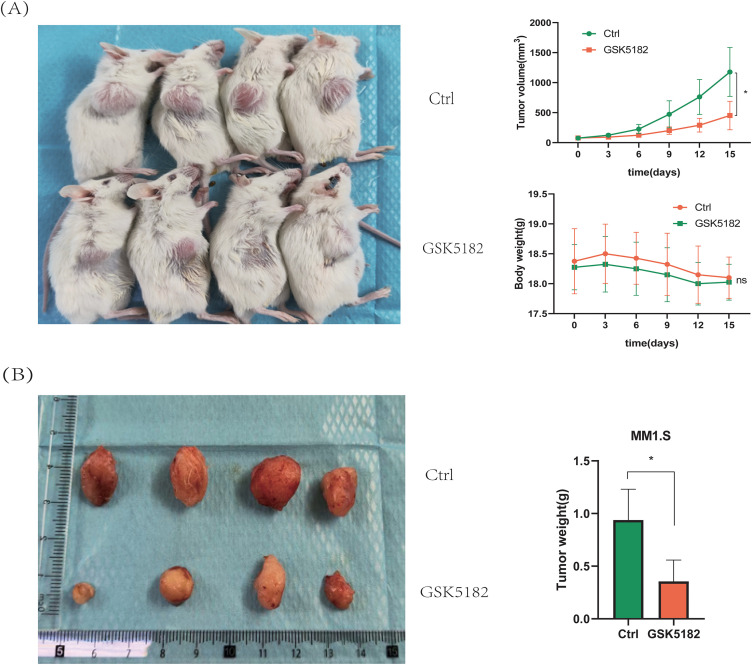
ERRγ inhibition suppresses MM tumor growth *in vivo*. **(A)** Tumor volumes and body weights were measured every 3 days during the 14-day GSK5182 treatment. **(B)** Tumors collected from GSK5182-treated and control groups. *p < 0.05, ns: not significant

Subcutaneous MM1.S xenografts were generated in NOD-SCID mice to investigate the anti-tumor effects of GSK5182 *in vivo*. Administration of GSK5182 (40 mg/kg/day, intraperitoneally) for 14 days led to a significant reduction in tumor volume compared to the vehicle-treated group, with tumor measurements recorded every three days (Fig. 4A). Further analysis confirmed a 62% decrease in tumor weight in the GSK5182-treated mice (Fig. 4B). Importantly, there were no significant changes in body weight throughout the treatment, indicating low systemic toxicity. These findings show that pharmacological inhibition of ERRγ successfully limits MM progression *in vivo* without major adverse effects.

### 3.4 ERRγ Inhibition Triggers Mitochondrial Dysfunction and Oxidative Stress

**Figure 5:**
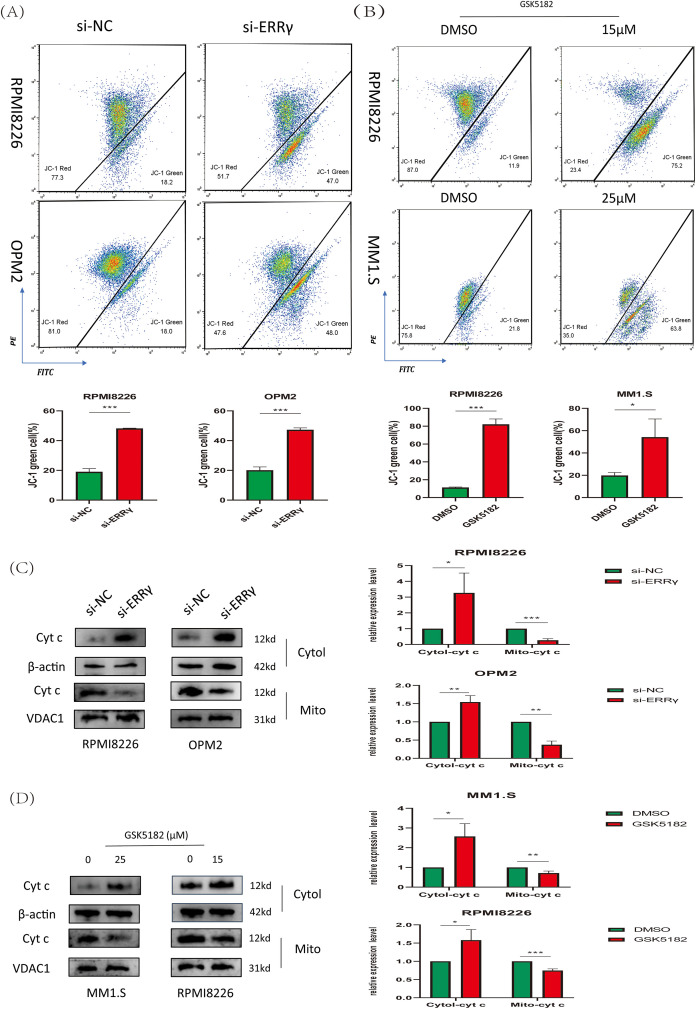
ERRγ inhibition triggers mitochondrial dysfunction. **(A,B)** JC-1 staining and flow cytometry analysis of mitochondrial membrane potential in MM cells following ERRγ knockdown or GSK5182 treatment for 48 h. Green fluorescence indicates a loss of mitochondrial membrane potential. **(C,D)** Western blot analysis was performed to assess the levels of cytochrome c in both cytoplasmic and mitochondrial fractions 48 h after the knockdown of ERRγ or treatment with GSK5182, revealing the release of cyt c into the cytoplasm. *p < 0.05, **p < 0.01, ***p < 0.001

The observed downregulation of BCL2 along with upregulation of BAX suggested that ERRγ inhibition might trigger apoptosis via the mitochondrial pathway. To explore mitochondrial dysfunction following ERRγ suppression, JC-1 staining and subcellular protein fractionation were conducted. In RPMI8226 and OPM2 cells, ERRγ knockdown by siRNA significantly increased the proportion of cells with green fluorescence, indicating a collapse of mitochondrial membrane potential (ΔΨm) (Fig. 5A). Similarly, treatment with GSK5182 (25 μM for MM1.S and 15 μM for RPMI8226) led to a substantial loss of ΔΨm in both MM1.S and RPMI8226 cells, as assessed by flow cytometry (Fig. 5B). The loss of ΔΨm typically results in the release of cytochrome c (Cyt c) from mitochondria into the cytoplasm [18]. Western blot analysis confirmed Cyt c downregulation in mitochondrial protein fractions and upregulation in cytoplasmic protein fractions (Fig. 5C,D). These results indicate that ERRγ inhibition compromises mitochondrial integrity, initiating Cyt c-mediated apoptosis.

**Figure 6:**
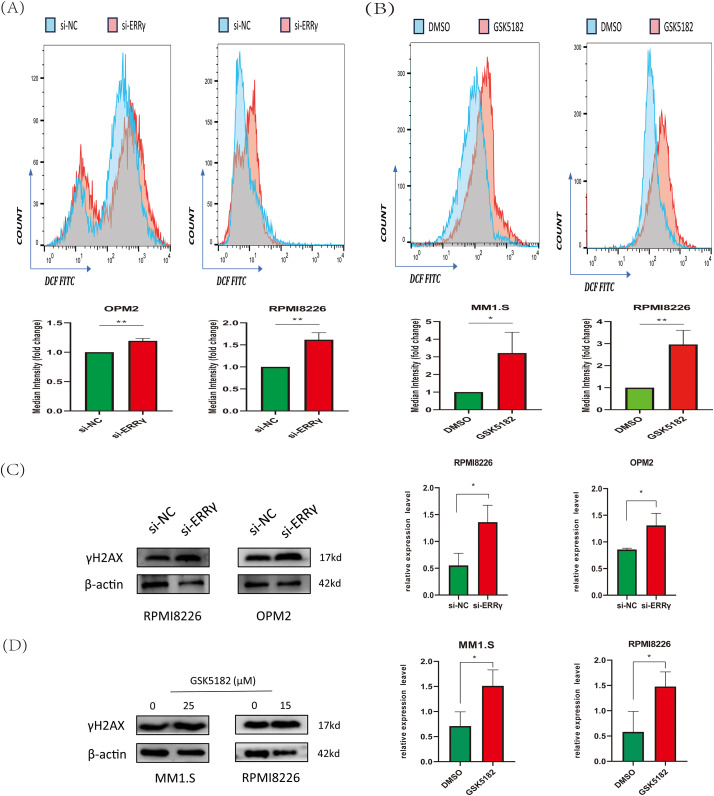
ERRγ inhibition induces reactive oxygen species (ROS) accumulation and DNA damage. **(A,B)** Flow cytometry analysis of 2^′^,7^′^-Dichlorodihydrofluorescein diacetate (DCFH-DA) fluorescence in MM cells 48 h post-ERRγ knockdown **(A)** or GSK5182 treatment **(B)**. **(C,D)** Western blot analysis of γH2AX expression in MM cells following ERRγ inhibition. *p < 0.05, **p < 0.01

Mitochondrial dysfunction is associated with increased reactive oxygen species (ROS) levels, which play crucial roles in regulating cell proliferation and apoptosis [19,20]. Flow cytometry analysis revealed a forward shift in DCFH-DA peaks in MM cells following ERRγ inhibition, suggesting increased intracellular ROS generation (Fig. 6A,B). Excessive ROS can cause oxidative damage to macromolecules, particularly DNA [21]. We evaluated the effect of ERRγ inhibition on γH2AX, a well-established marker of DNA damage. Both GSK5182 treatment and siRNA-mediated ERRγ knockdown elevated γH2AX levels in MM cells (Fig. 6C,D).

**Figure 7:**
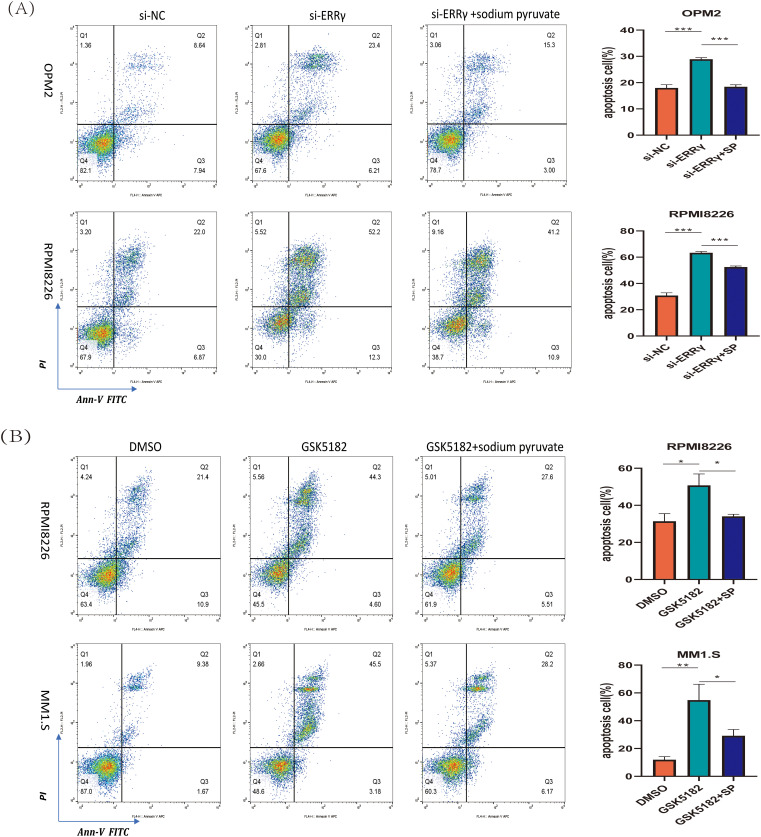

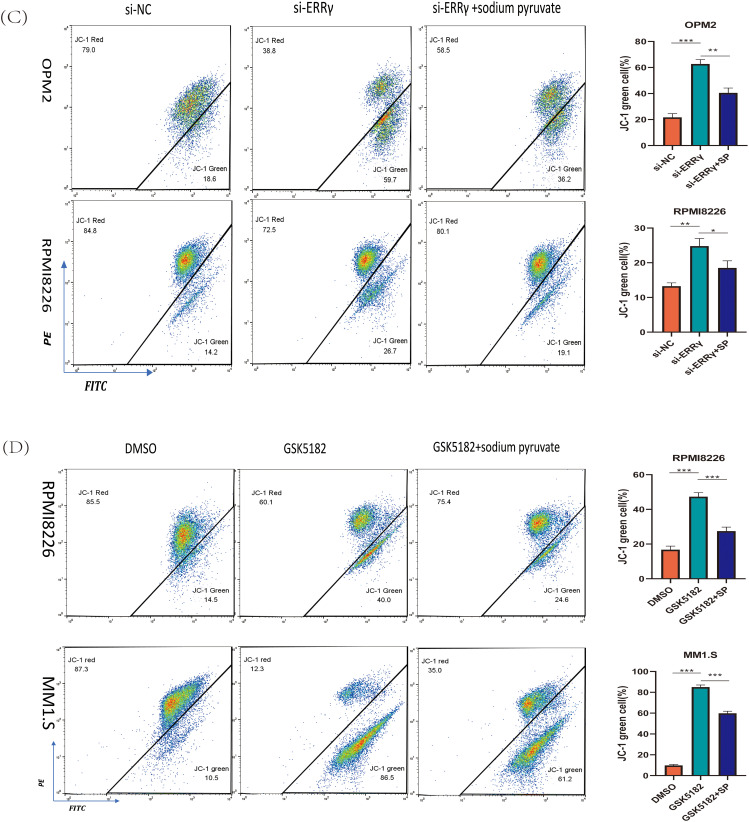
Sodium pyruvate (SP) partially rescues ERRγ inhibition-induced apoptosis. **(A,B)** Flow cytometry analysis of apoptosis in MM cells pretreated with 10 mM sodium pyruvate for 1 h, followed by ERRγ inhibition with siRNA or GSK5182 for 48 h. **(C,D)** Flow cytometry analysis of mitochondrial membrane potential in MM cells under the same conditions. *p < 0.05, **p < 0.01, ***p < 0.001

Sodium pyruvate (SP), a known antioxidant and tricarboxylic acid (TCA) cycle substrate, has been reported to ameliorate mitochondrial disorders [22,23]. To investigate the mechanisms driving apoptosis following ERRγ inhibition, MM cells were pretreated with 10 mM sodium pyruvate for 1 h before being subjected to ERRγ inhibition. Flow cytometry demonstrated that sodium pyruvate partially alleviated apoptosis induced by ERRγ suppression (Fig. 7A,B). In parallel, JC-1 staining showed that pretreatment with sodium pyruvate significantly decreased the proportion of cells with green fluorescence (Fig. 7C,D). These results identify the mitochondria-ROS-apoptosis axis as the primary pathway mediating the anti-myeloma effects of ERRγ inhibition.

### 3.5 ERRγ Sustains NF-κB Signaling in MM

**Figure 8:**
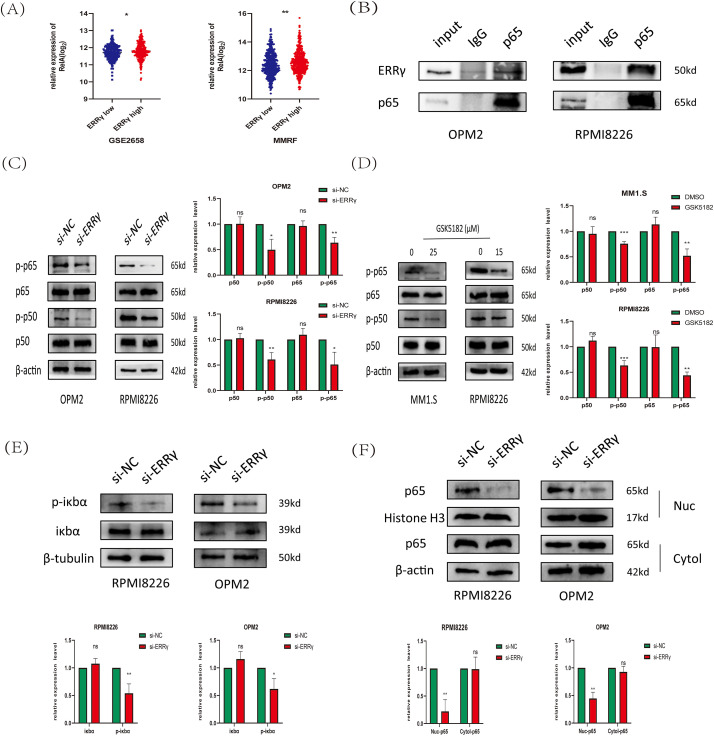
ERRγ regulates NF-κB pathway activation in multiple myeloma. **(A)** Gene expression analysis from MMRF and GSE2658 datasets showing higher RelA levels in plasma cells with elevated ERRγ. **(B)** Co-IP assays confirming an endogenous interaction between ERRγ and p65 in MM cell lysates. **(C)** Western blot showing decreased phosphorylation of p65 and p50 in OPM2 and RPMI8226 cells after ERRγ knockdown. **(D)** MM1.S and RPMI8226 cells treated with GSK5182 (25 and 15 μM, respectively) also exhibited reduced p65/p50 phosphorylation. **(E)** Western blot analysis of phospho-IκBα and total IκBα levels in ERRγ-silenced OPM2 and RPMI8226 cells. **(F)** Western blot of nuclear and cytoplasmic fractions demonstrating reduced nuclear p65 accumulation following ERRγ knockdown. *p < 0.05, **p < 0.01, ***p < 0.001; ns: not significant

Previous research suggests that ERRγ interacts with p65 in breast cancer to regulate ABCB1 expression [8]. Moreover, the NF-κB pathway has been reported to directly regulate mitochondrial function [24]. To explore the association between ERRγ and NF-κB signaling in multiple myeloma, public datasets (MMRF and GSE2658) were analyzed, revealing significantly higher RelA gene expression in plasma cells with elevated ERRγ levels (Fig. 8A). Co-immunoprecipitation (Co-IP) experiments confirmed a direct binding between ERRγ and p65 in MM cells (Fig. 8B). Functional studies showed that siRNA-mediated ERRγ knockdown in OPM2 and RPMI8226 cells, or pharmacological inhibition with GSK5182 in MM1.S and RPMI8226 cells, led to reduced phosphorylation of p65 and p50 (Fig. 8C,D), suggesting impaired NF-κB pathway activity. As the canonical NF-κB pathway relies on IκBα phosphorylation for p65 nuclear translocation [25]. Western blot analysis demonstrated that ERRγ silencing reduced phospho-IκBα levels (Fig. 8E), and nuclear protein isolation revealed reduced nuclear localization of p65 (Fig. 8F). Together, these results indicate that ERRγ facilitates NF-κB activation by promoting IκBα phosphorylation, p65/p50 activation, and p65 nuclear translocation.

### 3.6 ERRγ May Mediate Bone Destruction by Regulating RANKL Expression

**Figure 9:**
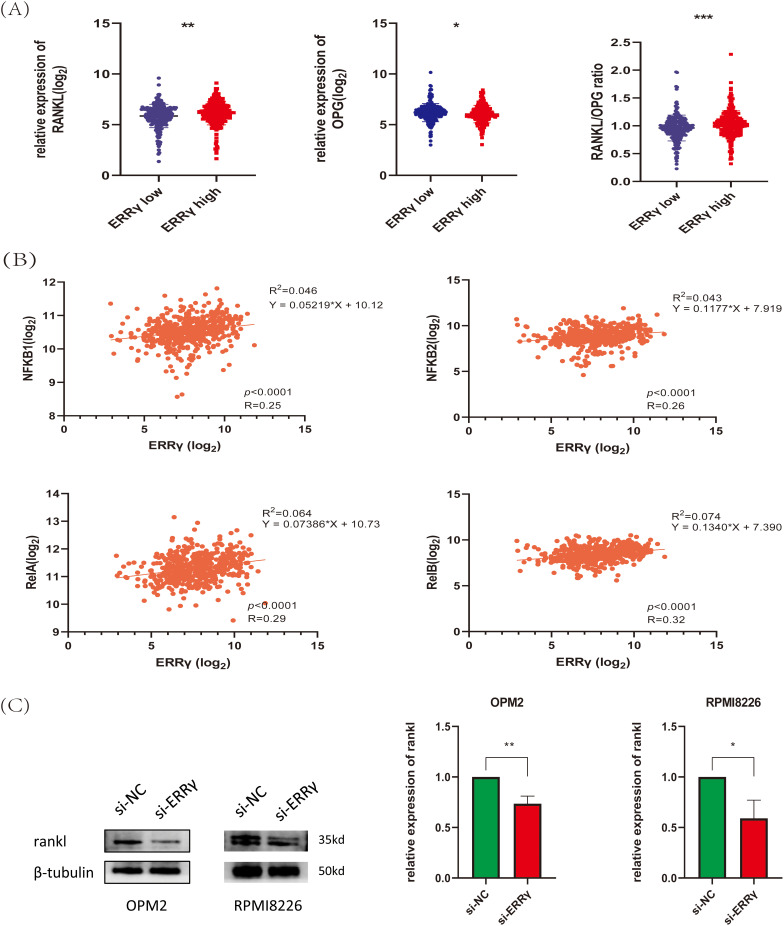

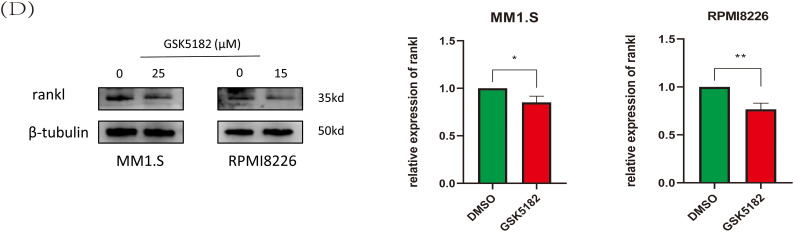
ERRγ may mediate bone destruction by regulating RANKL expression. **(A)** Bone marrow biopsy data from the GSE118985 dataset show that higher ERRγ expression is linked to an increased RANKL/OPG ratio. **(B)** Correlation analysis reveals a positive association between ERRγ expression and NF-κB pathway genes (NFKB1, NFKB2, RelA, and RelB). **(C,D)** Western blot results showing reduced RANKL protein levels in MM cells after ERRγ knockdown or GSK5182 treatment for 48 h. *p < 0.05, **p < 0.01, *** *p* < 0.001

Research has demonstrated that MM cells secrete RANKL, which promotes its expression in bone marrow stromal cells. RANKL then binds to RANK receptors on osteoclasts, activating NF-κB signaling and promoting bone degradation [14,26,27]. Analysis of the GSE118985 dataset (n = 460 newly diagnosed MM patients) revealed that bone marrow biopsies with high ERRγ expression exhibited elevated RANKL (pro-osteoclastogenic factor) and reduced OPG (osteoclastogenesis inhibitor) levels. Furthermore, ERRγ expression positively correlated with genes involved in the NF-κB pathway (NFKB1, NFKB2, RelA, RelB) (Fig. 9A,B), suggesting coordinated transcriptional regulation. Supporting these observations, inhibition of ERRγ reduced RANKL protein expression in MM cell lines (Fig. 9C,D). These findings suggest that ERRγ might be involved in regulating the RANKL/NF-κB axis, contributing to bone destruction in multiple myeloma.

## 4 Discussion

This study identifies Estrogen-Related Receptor Gamma (ERRγ) as a clinically relevant oncogene in multiple myeloma (MM), with its elevated expression associated with advanced disease stages and osteolytic bone damage. Suppression of ERRγ was shown to inhibit MM progression both *in vitro* and *in vivo* by promoting mitochondrial-mediated apoptosis and interfering with NF-κB signaling. Additionally, ERRγ may influence MM-related bone destruction by regulating the RANKL/NF-κB signaling pathway. Collectively, these results suggest that ERRγ represents a promising therapeutic target in MM.

Mitochondria are the primary physiological source of reactive oxygen species (ROS), and their dysfunction leads to increased oxidative stress [28]. Our data demonstrate that ERRγ knockdown or pharmacological inhibition induces mitochondrial membrane potential collapse, cytochrome c release, and an elevated BAX/BCL-2 ratio, consistent with intrinsic apoptosis activation. These mitochondrial perturbations coincide with ROS accumulation, indicating that ERRγ loss disrupts mitochondrial integrity and redox balance. The partial rescue of ΔΨm and apoptosis by sodium pyruvate—a TCA cycle substrate known to replenish mitochondrial metabolites and mitigate membrane permeability transition [29]—confirms that ERRγ inhibition-induced cytotoxicity is mechanistically linked to mitochondrial metabolic failure. These observations align with prior studies establishing ERRγ as a master regulator of mitochondrial metabolism and redox balance [30,31]. Taken together, our data indicate that ERRγ is vital for preserving mitochondrial integrity and metabolic homeostasis in MM cells, and that its inhibition disrupts these processes, thereby activating intrinsic apoptosis.

Emerging evidence highlights the multifaceted role of NF-κB pathway components in mitochondrial function and apoptosis regulation. Notably, p50, p65, and IκBα have been localized to mitochondria, where they modulate mitochondrial biogenesis, oxidative phosphorylation, and apoptosis [32,33]. For instance, IκBα interacts with VDAC and mitochondrial HKII at the outer mitochondrial membrane to stabilize the VDAC-HKII complex, thereby inhibiting Bax-mediated cytochrome c release and apoptosis [34]. Conversely, NF-κB inhibition in macrophages reduces BCL2 expression, disrupts mitochondrial membrane potential, and triggers mitochondrial apoptosis [35], underscoring the pathway’s context-dependent roles in cell survival. Our data suggest that ERRγ coordinates NF-κB signaling and mitochondrial apoptosis regulation in MM. In patient cohorts, elevated ERRγ expression correlated with upregulated P65, and co-immunoprecipitation confirmed a direct interaction between ERRγ and P65. Mechanistically, ERRγ inhibition attenuated phosphorylation of P65 and p50, suppressed P65 nuclear translocation, and perturbed the canonical NF-κB pathway. These effects were accompanied by increased BAX expression, decreased BCL2 levels, ΔΨm collapse, and cytochrome c release in MM cells, suggesting that ERRγ exerts its pro-survival effects, at least in part, through NF-κB-mediated mitochondrial apoptosis regulation. This aligns with studies showing NF-κB’s dual role in mitochondrial metabolism and apoptosis [36].

While the NF-κB pathway is essential for MM progression, direct inhibition often leads to systemic toxicity because of the pathway’s broad role in immune and inflammatory regulation [37]. In contrast, our data suggest that GSK5182, a selective ERRγ inverse agonist, attenuates NF-κB activity in MM cells without reducing body weight in treated mice, a preliminary but consistent indicator of tolerability. Furthermore, studies in non-tumor models suggest GSK5182 may have protective effects in acute liver injury and asthma [38,39]. Together, these findings imply that targeting ERRγ could offer a potentially safe therapeutic approach for MM, although extensive toxicological studies are necessary to confirm its clinical applicability.

A hallmark of multiple myeloma is pathological osteoclast activation driven by RANKL/NF-κB signaling, which fuels osteolytic bone destruction [26,40,41]. Our data reveal that ERRγ expression is significantly elevated in MM patients with pathological fractures. Bioinformatic analysis of publicly available datasets demonstrates that high ERRγ levels correlate with RANKL upregulation, OPG suppression, and NF-κB pathway activation in the bone marrow. These clinical correlations align with *in vitro* evidence: ERRγ inhibition in MM cells reduces RANKL secretion, implying its regulatory influence on RANKL production. While causality remains unproven, collective data suggest that ERRγ may amplify RANKL/NF-κB signaling in the bone microenvironment, a hypothesis partially supported by studies in non-MM contexts showing ERRγ inhibition suppresses RANKL-mediated osteoclastogenesis [10] and parallels its role in NF-κB-driven bone remodeling in osteoarthritis [42]. Importantly, these observations position ERRγ as a therapeutic target with dual relevance—its inhibition could not only impair MM cell survival but also theoretically attenuate RANKL-driven osteoclast activation, though direct evidence of bone protection in MM models is still needed.

Although our findings establish ERRγ as a key oncogenic driver in MM, this study has two main limitations. First, while ERRγ inhibition reduced tumor burden in xenograft models, its molecular mechanism remains undefined, and systematic toxicity profiling of GSK5182 remains incomplete. Second, although our data suggest that ERRγ modulates RANKL to promote osteolysis, direct evidence linking ERRγ to osteoclast activation and *in vivo* bone protection is lacking. Future studies will focus on elucidating ERRγ’s regulatory role in the bone microenvironment.

In summary, our study provides initial insights into the potential role of ERRγ in MM. We demonstrate, for the first time, that ERRγ is upregulated in MM cells and may promote disease progression by modulating NF-κB signaling and maintaining mitochondrial function. In addition, ERRγ may contribute to MM-associated bone destruction through the regulation of RANKL expression. These findings highlight the therapeutic potential of targeting ERRγ in the treatment of MM.

## Data Availability

The datasets used during the present study are available from the corresponding author upon reasonable request.

## Abbreviations

MM: Multiple Myeloma
ERRγ: Estrogen-Related Receptor Gamma
RANKL: Receptor Activator of Nuclear Factor-κB Ligand
NF-κB: Nuclear Factor-Kappa B
siRNA: Small Interfering RNA
IC_50_: Half-Maximal Inhibitory Concentration
SP: Sodium Pyruvate
TCA: Tricarboxylic Acid
IDA: Iron-deficiency Anemia
ΔΨm: Mitochondrial Membrane Potential
ROS: Reactive Oxygen Species
Cyt c: Cytochrome c
